# Feasibility, acceptability and potential helpfulness of the PROACTIVE intervention in Flanders, Belgium: A survey study

**DOI:** 10.1371/journal.pone.0289952

**Published:** 2023-08-10

**Authors:** Shanshan Wang, Johanna de Almeida Mello, Mary S. Mittelman, Anja Declercq

**Affiliations:** 1 LUCAS–Centre for Care Research & Consultancy, KU Leuven, Leuven, Belgium; 2 Department of Oral Health Sciences, Research Group Population Studies in Oral Health, KU Leuven, Leuven, Belgium; 3 Department of Psychiatry, New York University School of Medicine, New York, NY, United States of America; 4 CeSO—Centre for Sociological Research, KU Leuven, Leuven, Belgium; University of Sharjah, UNITED ARAB EMIRATES

## Abstract

**Background/objectives:**

This study aimed to explore the feasibility of a Flemish adaptation of the New York University Caregiver Intervention (i.e., PROACTIVE intervention) modifying the recruitment and intervention content for informal caregivers of people with early cognitive decline, and across different subgroups. A feasibility study is necessary in order to reduce research waste for intervention adaptation and evaluation.

**Methods:**

Researchers constructed, tested, and sent out a survey consisting of 43 questions on the following topics: awareness of symptoms of early cognitive decline, levels of cognitive performance using the updated Cognitive Performance Scale (CPS2), acceptability, and potential helpfulness of the intervention, and sociodemographic characteristics. Quantitative data were analyzed using descriptive statistics and logistic regression with SAS 9.4©. Qualitative data were analyzed using an inductive content analysis.

**Results:**

A total of 463 informal caregivers completed the survey (mean age 58.8 ± 11.8, 83.6% female). Among them, 230 respondents who cared for people with cognitive decline indicated they would probably or certainly participate in the study. Identified factors influencing the recruitment were cognition, co-habitation, education, and employment status. Over half of the target caregivers indicated almost all services from the intervention could satisfy their needs. A majority perceived the PROACTIVE intervention would be helpful (69.4%), especially the CPS2 = 3 (76.1%) and CPS2 = 4 (74.1%) subgroups.

**Conclusion:**

The recruitment of target participants for a subsequent RCT evaluation study is feasible, and identified associated factors should be considered during the recruitment process. The PROACTIVE intervention and core components except ‘peer-group participation’ were perceived as helpful by most caregivers. The CPS2 = 3–4 subgroups were most accepting of the intervention and were most likely to benefit from the intervention.

## Introduction

According to the 2019 World Population Prospects, 16% of the world population will be over age 65 and 426 million people will be over age 80 by 2050 [1]. Given the projected trends in population ageing, the number of people with dementia will increase to 152.8 million cases in 2050 [2]. This will cause an increasing demand for healthcare resources and a threat to sustainable healthcare [3]. The number of people with dementia (young-onset dementia included) in Flanders, Belgium, was estimated to increase by more than 40% by 2035 compared to 2018, which was estimated at 131,818 [4, 5]. A recent longitudinal cohort study highlighted the problem of ageing population using a representative sample in general practice for the Flemish population, Belgium. This study showed a significantly increasing trend in the prevalence of registered dementia cases and of registered multimorbidity (3+ comorbidities) in dementia patients over from 2000 to 2021 [6]. Providing early support for informal caregivers can improve their wellbeing and ability to care, keeping older people with cognitive decline at home longer and diminishing public healthcare costs [7–11]. However, scientific literature has shown that very few psychosocial interventions target informal caregivers at the early stage of caregiving [11, 12]. In Belgium, a nationwide research project showed that informal caregivers of older people with early cognitive decline perceived burden and were at risk of discontinuing care at home [13].

The New York University Caregiver Intervention (NYUCI) aims to improve informal caregivers’ wellbeing and thereby enable them to keep caring for the person with dementia at home longer. The NYUCI is a multicomponent intervention that includes a comprehensive assessment of the caregiver, followed by an individual counseling session, four family counseling sessions, and then a second individual session within four months of enrollment. Participation in a support group is recommended. Additional counseling, called “ad hoc” counseling, is available to caregivers and participating families as needed. The NYUCI has been widely replicated and implemented in various locations across the United States and in several other countries over the past 30 years. The intervention was developed and evaluated in a randomized controlled trial funded by the National Institutes of Health from 1987 to 2010. Published research demonstrated that the NYUCI showed multiple long-term benefits for both family caregivers and individuals with dementia, including improved social support and self-rated health, reduced depression and reactions to problem behaviors, postponement of residential care placement and major healthcare cost savings [14–20]. The improvement of key outcomes (e.g., caregiver burden, symptoms of depression) were also achieved among adult child caregivers, although the NYUCI was originally designed for spousal caregivers [21, 22].

The PROACTIVE intervention will be the first Flemish adaptation of the NYUCI focusing specifically on informal caregivers of people with early cognitive decline, compared to the NYUCI which includes caregivers of people at all stages of dementia [23]. In Flanders, we will focus on caregivers of people at the early stage of cognitive decline, as these caregivers often receive no help or support at the beginning of their caregiving activities and are often left out of formal help services. Evidence also shows that the target population of our intervention are often hard to reach, because these informal caregivers struggle with self-identification as caregivers, identifying and acknowledging needs and with accepting help [24, 25]. Moreover, effects of an intervention are highly dependent on the context. To reduce research waste and successfully replicate effects of the NYUCI, a careful and systematic adaptation process improves the likelihood of achieving a good intervention-context fit [26]. A brief description of the planned PROACTIVE intervention was mentioned in our protocol [23]. Potential barriers to success include low acceptability of the intervention, difficulties with recruitment capability and retention, as well as difficulties with convincing participants to complete questionnaires necessary to evaluate the intervention. A feasibility study can provide information about how to overcome these barriers, especially for these vulnerable hard-to-reach participants [27, 28].

The UK Medical Research Council (MRC) provides a framework for developing and evaluating complex interventions, including the identification or development of an intervention, and the feasibility, evaluation and implementation phases. Each phase should consider the following core elements: context, program theory, stakeholders, key uncertainties, intervention refinement, and cost-effectiveness [29]. Using the MRC framework, this study has the objective of exploring the feasibility of the PROACTIVE intervention, including the feasibility of recruiting participants and the adequacy and helpfulness of the intervention content. Specifically, we wish to answer the following questions: 1) Can we recruit enough participants for the evaluation of the PROACTIVE intervention, and what barriers should we address when conducting this recruitment? 2) Can the intervention content meet the needs of the target participants and are there different needs among subgroups stratified by the level of cognitive performance (acceptability of the intervention content)? 3) Are the intervention and its core components perceived as helpful by our the target participants and across subgroups stratified by the level of cognitive performance (potential helpfulness of the intervention)?

## Methods

The study was approved by the Ethics Committee of the KU Leuven under the number G-2020-1771-R2(MAR). The collection and processing of personal data meets the requirements of the General Data Protection Regulation (GDPR) confirmed by KU Leuven’s Data Protection Officer (‘DPO’). At the beginning of our anonymous online survey, we clearly state the respondent’s right to confidentiality, possible risks and benefits from participating in our study. Informed consent was also obtained from all participants. The reporting of this survey follows the Checklist for Reporting of Survey Studies (CROSS) [30].

### Study design and recruitment

The survey was programmed in Qualtrics©. An anonymous link was provided to respondents and no personally identifiable information was collected in the survey. The survey was opened in February 2022 and closed in June 2022. Eligible participants were informal caregivers who were aged 18 years or older, and who provided support to a community-dwelling person who had cognitive problems. In this study, informal caregivers were defined as family members, siblings, other relatives, friends, or neighbors. They could be primary caregivers or secondary caregivers.

Participants were recruited through newsletters of organizations (e.g. memory clinics; Belgian Alzheimer centers, etc.), via social media posts (e.g. posts in Facebook groups), or by recommendation from physicians. We also recruited participants from organizations working with older people with cognitive decline (day care centers, home care organizations). We encouraged all the respondents to share the link to the survey within their networks (e.g. family, friends). Most informal caregivers (93%) filled out the survey online, while some informal caregivers (7%) filled out the survey on paper. No remuneration was offered for participation in this study.

### Outcome measures

The survey was constructed by the research team based on the intended benefits of the NYUCI components, evaluation forms and questionnaires from the NYUCI, and knowledge about the symptoms of early cognitive decline. Eligible informal caregivers whose relative or friend or neighbor had cognitive problems filled out the survey which included their rating of the individual’s level of cognitive performance through the items from the updated interRAI Cognitive Performance Scale (CPS2) [31]. The survey was programmed in the Qualtrics© software application. The survey underwent three rounds of pretesting with the same potential participants. The number of potential participants for pretesting was seven. These potential participants were recruited in the network of the researchers’ friends, parents, grandparents or neighbors. Pretesting data were collected through the seven test participants’ completion of the online survey which was programmed in the Qualtrics© software application. During the pretesting process, we changed some professional and technical/clinical wording into familiar language more appropriate for participants’ age, education, and experiences. We also corrected some spelling mistakes after some additional adaptations, such as language formulation to improve content comprehensibility until a final version was approved by the research team. The 43 questions included in the survey covered the following domains: awareness of symptoms of the individual’s cognitive decline, level of the individual’s cognitive performance, acceptability and potential helpfulness of the PROACTIVE intervention, willingness to participate in the PROACTIVE study, and sociodemographic characteristics.

The first module of the survey contained six questions with a 5-point Likert scale (1 = never, 2 = occasionally, 3 = sometimes, 4 = often, 5 = always) on the respondents’ awareness of the individual’s symptoms of early cognitive decline. Questions about symptoms of cognitive decline include ‘repeating or asking the same thing over and over’, ‘hard to follow conversations’, ‘confused about time and place’. We also included the question ‘Do you agree your relative’s or friend’s or neighbor’s symptoms are a natural part of ageing?’ in a 5-point Likert scale (1 = strongly agree, 2 = agree, 3 = neutral, 4 = disagree, 5 = strongly disagree).

The second module of the survey consisted of the updated Cognitive Performance Scale (CPS2) which assesses the caregiver’s perception of their relative’s or friend’s or neighbor’s six domains of cognition: short-term memory (Yes/No), making self-understood (5 scoring levels), cognitive skills for daily decision-making (6 scoring levels), managing finances (7 scoring levels), managing medications (7 scoring levels), and walking (8 scoring levels). Mini-Mental State Examination (MMSE) has been mandatorily used in Belgium, and a study found the MMSE and CPS have almost similar performance for cognitive impairment test in nursing home residents [32]. Compared to the original CPS (range 0 to 6), a study demonstrated that the CPS2 scale (range 0 to 8), when used in repeated assessments, is sensitive to detect changes particularly in early levels of cognitive decline [31]. Another study explored the score conversion between Montreal Cognitive Assessment and CPS and CPS2 using a large cohort of older people with mild physical and cognitive impairment. It showed that the highest scores observed were 3 for CPS and 5 for CPS2 in this mild impairment sample [33]. According to Morris JN, et al. (2016), the scores of the CPS2 scale have the following descriptions: 0 = intact1, 1 = intact2, 2 = borderline1, 3 = borderline2, 4 = moderately impaired1, 5 = moderately impaired2, 6 = severe1, 7 = severe2 and 8 = very severe impairment. In this study, we defined ‘early cognitive decline’ as equivalent to a CPS2 score of 1–4 and we selected our target population based on this CPS2 selection criteria throughout this research.

Acceptability refers to the extent which the intervention components can meet the needs of the target population and how well the intervention can be delivered to the target population (e.g., acceptability of intervention delivery methods and tools) [34]. In the third module, we included 14 questions on the services the PROACTIVE intervention would provide, to be rated on a 5-point scale (1 = not applicable, 2 = satisfied, 3 = low need, 4 = need, 5 = high need). The 14 questions were designed to explore if the intervention content could satisfy or meet the needs of our target population, namely the acceptability of the intervention content. These services covered five domains: coping with changes (in relationship, in behavior or changes to be expected in the future) (4 items), informational support for informal caregivers or care recipients (2 items), talking to a professional counselor (2 items), peer support group participation (1 item), support from family members and family meetings (5 items). In the next preparation phase of the intervention adaptation, we will further explore the acceptability of the intervention delivery (e.g., delivery format).

In the fourth module, the potential helpfulness of the entire PROACTIVE intervention (1 item) and the components of the intervention (4 items) were explored using a 5-point scale (1 = not at all helpful, 2 = a little helpful, 3 = helpful, 4 = very helpful, 5 = extremely helpful). These five questions allowed us to explore to what extent the participants think the intervention, and each of the intervention components would be helpful. The last question of the module was the following: ‘Would you want to participate in the PROACTIVE study?’ (1 = not at all, 2 = possibly, 3 = probably, 4 = certainly). In the beginning part of the survey, the goal of the research is explained and there is a mention of the randomized controlled trial to evaluate the effectiveness of the PROACTIVE intervention in future. Participants were informed that there would be an experimental group and a control group. Therefore, this question allowed us to investigate whether we could expect sufficient recruitment for the RCT evaluation study and to identify factors associated with their willingness to participate the PROACTIVE intervention.

The last section contained ten questions about sociodemographic characteristics. The PROACTIVE intervention will be developed by adapting the NYUCI to the context of Flanders, one of the three Belgian regions. The survey was written and completed in Dutch, so respondents were mostly from the Flanders, Belgium. The recruitment was limited to the informal caregivers who live in the Flanders region. The adaptation phase of the intervention will include people from other ethnicities, who will also be involved in the stakeholders’ group. In this section, we asked questions about age, gender, highest level of education attained, relationship status, employment status, caring for children or others, co-habitation with the person with cognitive decline (defined as living together for at least 6 months), frequency of contact with the person with cognitive decline, relationship with the person with cognitive decline. The survey ended with an open-field question to welcome any feedback or thoughts from the respondents.

### Statistical analysis

First, a descriptive analysis was performed on the total sample and on subgroups stratified by the extent of willingness to participate in the PROACTIVE study. We included all levels of cognitive performance (CPS2 = 1–8) in the descriptive analysis and ordinal regression analysis, in order to get a whole picture of our sample around their willingness to participate in the PROACTIVE study and associated factors (e.g., level of cognitive performance). Ordinal regression analysis was then performed to identify factors associated with the willingness to participate in the intervention through modeling the possible determinants. We chose ordinal regression analysis to be able to predict the likelihood of our dependent outcome considering several predictors. We started with a full model and conducted a backward stepwise variable selection method until the significant determinants were retained. Our backward ordinal logistic model was built with the following variables: age = 60+, gender, relation to the person cared for, marital status, having children, help for caring for own children or other dependents, employment status, education level, co-habitation, level of cognitive performance. The 95% confidence interval (CI) was used to estimate the precision of the odds ratio (OR). OR>1 means higher odds of outcome, and OR < 1 lower means lower odds.

In addition, a descriptive analysis was also performed to identify the potential helpfulness and acceptability of the PROACTIVE intervention among the informal caregivers of older people with early cognitive decline (CPS2 = 1–4). A one-way ANOVA analysis was then performed to explore if there were significant differences in helpfulness and acceptability of the PROACTIVE intervention across subgroups stratified by the level of cognitive performance (CPS2 = 1,2,3 and 4). We did not perform Bonferroni correction, considering that our study is exploratory involving post-hoc testing [35]. Potential helpfulness of the PROACTIVE intervention was defined as the combination of responses ‘helpful, very helpful, extremely helpful’. Acceptability of the PROACTIVE intervention was defined as the combination of responses ‘low need’, ‘need’, ‘high need’ of the five domains of services which the PROACTIVE intervention provides. The 5-point scale (1 = not at all helpful, 2 = a little helpful, 3 = helpful, 4 = very helpful, 5 = extremely helpful; 1 = not applicable, 2 = satisfied, 3 = low need, 4 = need, 5 = high need) was dichotomized to help us better explore the differences in the acceptability and helpfulness of the intervention across subgroups, stratified by the level of cognitive performance. We summed 3 of them (3,4,5) and interpreted it as a positive response to explore more possibilities and obtain a broader view of the acceptable and helpful intervention content. Quantitative data analyses were conducted in SAS© software version 9.4.

Qualitative data was analyzed by means of a inductive content analysis. Factors, including translator, back-translation, culture, and language, influence the quality of translation during cross-language qualitative data analysis [36]. During data preparation, the collected data in the source language (i.e., Dutch) was translated to the target language (i.e., English) with a focus on maintaining the integrity of the original content. One researcher analyzed the data in the target language (i.e., English). Another bilingual researcher proficient in both languages analyzed the data in the source language (i.e., Dutch). In accordance with the research questions, words of the given text responses were independently classified into much smaller content categories (i.e., open coding, creating categories and abstraction) by two researchers while keeping in mind the context for meaning of the responses [37]. The written words of the given text response were read through several times. The two researchers came to a decision as to which things to put in the same category. Each researcher independently worked on the data analysis, and any discrepancies or differences were discussed and resolved through collaboration.

## Results

The survey questions were programmed in the Qualtrics© software application and participants were not allowed to skip certain questions. Only data from participants who filled out all questions of the survey were used for analysis. Among the total participants who filled out the survey, 40 participants didn’t fill out all the questions and stopped the survey. Thus, a total of 463 people completed all questions of the survey. We assumed that people who were interested in an intervention would be more inclined to fill out the survey. Most of them (355, 76.7%) became aware of the survey through social media (online groups about informal care, dementia, etc.), others through the newsletter of an organization (26, 5.6%), or via professionals or care organizations (e.g. home care services) (82, 17.7%). In the whole sample, the most frequently perceived cognitive problems were ‘Forgetting recent events or objects’ (347, 74.9%), and ‘Asking the same thing over and over’ (343, 74.1%). Half of the whole sample agreed with the statement that ‘Memory problems are a normal part of ageing’ (204, 44.1%). A total of 230 respondents (49.7%) reported that they would probably or certainly participate the PROACTIVE study, and 182 respondents (39.3%) reported that they would possibly participate the study.

As shown in Table 1, the mean age of the respondents was 58.8 (SD = 11.8, range 21–97), with 83.6% being female. Most of the respondents were married (74.7%) and had at least one child (77.5%—mean number of children: 1.7, SD = 1.2). About 84.4% of the respondents had almost daily contact with the individual with cognitive problems. Almost half of respondents were in the < = 60 age group (57.2%) and had to care for their own children or others (47.5%) and about 40.2% lived with the individual with cognitive problems. Adult children made up 29.4% of the total sample, while spouses/partners, parent/guardian and others (e.g., friend) made up 32.0%, 27.0% and 11.6% respectively. Regarding the representativeness of our sample, a comparison was made with a larger nationwide study on informal caregivers of frail older people (N = 11,363). In that nationwide sample, the median age of primary caregiver was 59 and about 38.5% lived with frail older people, which is similar to our sample (58.8, 40.2%). But in the nationwide sample, most primary caregivers of frail older people were adult children (55.5%), spouse and others made up respectively 30.6% and 13.9% [13].

**Table 1:** Profile of the whole population and subgroups stratified by the extent of willingness to participate the PROACTIVE study.

Respondents	Total = 463 n (%)	To what extent would you like to participate the PROACTIVE study			
		Not at all = 51 n (%)	Possibly = 182 n (%)	Probably = 146 n (%)	Certainly = 84 n (%)
Age group					
< = 60 (*Min* = 21)	265 (57.2)	23 (45.1)	102 (56.0)	82 (56.2)	58 (69.0)
>60 (*Max* = 97)	198 (42.8)	28 (54.9)	80 (44.0)	64 (43.8)	26 (31.0)
Age mean (*SD*)	58.8(11.8)	62.2(9.4)	59.5 (11.6)	58.6 (12.8)	55.6(10.9)
Gender (Female)	387 (83.6)	42 (82.4)	147 (80.8)	126 (86.3)	72 (85.7)
Married/Partner	346 (74.7)	39 (76.5)	138 (75.8)	106 (72.6)	63 (75.0)
Number of children mean (*SD*)	1.7 (1.2)	1.9 (1.0)	1.8 (1.2)	1.7 (1.2)	1.6 (1.2)
Number of children > = 1	359 (77.5)	45 (88.2)	144 (79.1)	118 (80.8)	63 (75.0)
Have almost daily contact with the person with cognitive decline (Yes)	391 (84.4)	44 (86.3)	155 (85.2)	117 (80.1)	75 (89.3)
Live with the person with cognitive decline (Yes)	186 (40.2)	23 (45.1)	64 (35.2)	60 (41.1)	39 (46.4)
Help with childcare or have other dependents (Yes)	220 (47.5)	27 (52.9)	81 (44.5)	59 (40.4)	53 (63.1)
Relationship to the person with cognitive decline					
Child/Child-in-law	136 (29.4)	13 (25.5)	51 (28.0)	46 (31.5)	26 (31.0)
Spouse/Partner	148 (32.0)	18 (35.3)	52 (28.6)	51 (34.9)	27 (32.1)
Parent/Guardian	125 (27.0)	13 (25.5)	60 (33.0)	31 (21.2)	21 (25.0)
Others (e.g. sibling, friend, neighbor)	54 (11.6)	7 (13.7)	19 (10.4)	18 (12.3)	10 (11.9)
Education					
Middle school and others	125 (27.0)	24 (47.0)	50 (27.5)	35 (24.0)	16 (19.0)
Professional education	84 (18.1)	11 (21.6)	37 (20.3)	21 (14.4)	15 (17.9)
University or Higher education	254 (54.9)	16 (31.4)	95 (52.2)	90 (61.6)	53 (63.1)
Employment status					
Employed full time/Self-employed	126 (27.2)	7 (13.7)	41 (22.5)	41 (28.1)	37 (44.0)
Employed part time	115 (24.8)	13(25.5)	50 (27.5)	29 (19.9)	23 (27.4)
Retiree/Volunteer/Housewife	222 (47.9)	31 (60.8)	91 (50.0)	76 (52.0)	24 (28.6)
Levels of the individual’s cognitive performance (CPS2 = 1–8)					
Intact2 to Borderline1 (CPS2 = 1–2)	82 (17.7)	16 (31.4)	36 (19.8)	18 (12.3)	12 (14.3)
Borderline2 (CPS2 = 3)	138 (29.8)	10 (19.6)	63 (34.6)	39 (26.7)	26 (31.0)
Moderately impaired1 (CPS2 = 4)	58 (12.5)	6 (11.8)	23 (12.6)	18 (12.3)	11 (13.1)
Moderately impaired2 (CPS2 = 5)	61 (13.2)	3 (5.9)	22 (12.1)	28 (19.2)	8 (9.5)
Severe1 to severe2 (CPS2 = 6–7)	80 (17.1)	9 (17.6)	25(13.7)	30 (20.5)	16 (19.0)
Very severe (CPS2 = 8)	44 (9.6)	7 (13.7)	13 (7.1)	13 (8.9)	11 (13.1)

Across the four subgroups stratified by the extent of willingness to participate in the PROACTIVE study, from ‘Not at all’ to ‘Possibly’ to ‘Probably’ to ‘Certainly’, the mean age was 62.2, 59.5, 58.6, 55.6 respectively, and the mean number of children was 1.9, 1.8, 1.7, 1.6 respectively. The proportion of full time employed/self-employed respondents increased across the four subgroups along with the extent of willingness to participate the PROACTIVE study (from ‘Not at all’ to ‘Possibly’ to ‘Probably’ to ‘Certainly’: 13.7%, 22.5%, 28.1%, 44.0% respectively). This trend was also observed in respondents’ education with university or higher education (31.4%, 52.2%, 61.6%, 63.1%). The percentage of respondents who had a relative or friend or neighbor with intact2 to borderline1 cognitive performance (CPS2 = 1–2) followed the opposite pattern of willingness to participate (from ‘Not at all’ to ‘Possibly’ to ‘Probably’ to ‘Certainly’: 31.4%, 19.8%, 19.2%, 14.3%). Respondents who had a relative or friend or neighbor with borderline2 cognitive performance (CPS2 = 3) were most willing to participate in the PROACTIVE study (31.0%).

As shown in Table 2, results of the regression analysis showed that the most significant factor predicting willingness to participate was cognitive performance (OR:2.21, 95%CI: 1.41–3.48). It meant that compared to people who had a relative or friend or neighbor with intact2 to borderline1 cognitive performance, people who were caring for the individual with higher impaired level of cognitive performance also had an increased likelihood of willing to participate in the PROACTIVE study (i.e., from ‘not at all’ to ‘possibly’ to ‘probably’ to ‘certainly’). Three other significant factors were ‘Full-time employed/self-employed’ (OR:2.04, 95%CI: 1.27–3.29), ‘University or higher education’ (OR:2.04, 95%CI: 1.34–3.10), and ‘Living with the individual with cognitive problems’ (OR:1.64, 95%CI: 1.13–2.39).

**Table 2:** Ordinal regression analysis for factors associated with the extent of willingness to participate in the PROACTIVE study.

Predictors	OR	OR (95%-CI)	*P*
Cognitive performance (Ref = intact2 to borderline1, CPS = 1–2)	2.21	1.41–3.48	0.000***
Full-time employed/self-employed (Ref = part-time employed)	2.04	1.27–3.29	0.003**
University or higher education (Ref = lower education)	2.04	1.34–3.10	0.000***
Living with the individual with cognitive problems	1.64	1.13–2.39	0.009**
Gender (Ref = Male)	1.60	0.99–2.60	*0*.*056*

Notes: Grey numbers in Italic Font style also represent no significance, but were kept here due to the stopping criterion used in the backward logistic regression analysis. Significance:

***p < .001

**p < .01

*p < .05.

As shown in Table 3, in the total sample of people with early cognitive decline (CPS2 = 1–4, N = 278), a majority thought that all components of the PROACTIVE intervention would be helpful (69.4%). In their opinion, across the four intervention components, the most helpful was ‘Family meeting with a counselor’ (71.6%) and the least helpful was ‘Peer-support group participation’ (47.8%).

**Table 3:** Potential helpfulness of the PROACTIVE intervention by respondents’ relatives or friends or neighbors with early cognitive decline (CPS2 = 1–4, N = 278).

Potential helpfulness of intervention and its components	Total = 278 n (%)	CPS2 = 1 n (%)	CPS2 = 2 n (%)	CPS2 = 3 n (%)	CPS2 = 4 n (%)	*P*
Entire PROACTIVE intervention	193(69.4)	5(38.5)	40(58.0)	**105(76.1)**	43(74.1)	0.04*
Family meeting with a counselor	**199(71.6)**	4(30.8)	37(53.6)	110(79.7)	48(82.8)	0.000***
Talking to a professional counselor	194(69.8)	4(30.8)	39(56.5)	103(74.6)	48(82.8)	0.008**
Telephone/email consultation on request over the disease course	185(66.5)	3(23.1)	37(53.6)	102(73.9)	43(74.1)	0.01*
Peer-support group participation	133(47.8)	5(38.5)	27(39.1)	68(49.3)	33(56.9)	0.131

Notes: Ratings of these items were made on a 5-point Likert scale (1 = Not at all helpful, 2 = A little helpful, 3 = Helpful, 4 = Very helpful, 5 = Extremely helpful). ‘Helpfulness’ is based on summed values of ‘helpful, Very helpful, Extremely helpful’. Significance:

***p < .001

**p < .01

*p < .05.

Across the 4 subgroups of levels of cognitive performance, namely intact2, borderline1, borderline2 and moderately impaired1, the subgroup caring for someone with borderline2 cognitive performance (CPS2 = 3) mostly thought the entire intervention would be helpful (76.1%). In subgroups with borderline2 and moderately impaired1 (CPS2 = 3,4), participants thought the most helpful intervention component would be ‘Family meeting with a counselor’ (79.7%, 82.8% respectively). In the subgroup with borderline1 cognitive performance (CPS2 = 2), the most helpful intervention component would be “primary caregiver can talk to a professional counselor’ (56.5%).

As shown in Table 4, almost half of the people with early cognitive decline (N = 278) said they thought they needed all components of the intervention. Across all the 14 components, the most frequent needed component was related to help to adapt to future changes—‘Knowing what changes to expect and how to adapt to these changes’ (66.2%). Three other frequently reported needs were related to proactive planning for the future including ‘Proactive planning for future care planning, financial planning etc.’ (60.1%), regular help from a professional counselor (59.4%), and information accessibility and support for you as a caregiver (58.3%). Only three needs were reported by less than 50% of respondents in the sample with early cognitive decline (CPS2 = 1–4), namely ‘Other family members or friends or neighbors talk to a professional counselor’ (47.8%), ‘Knowing reliable information on the condition of the individual with cognitive problems’ (47.1%), and ‘Family meetings to talk about the condition, future plan, etc. of the individual with cognitive problems’ (47.1%).

**Table 4:** Needed services which the PROACTIVE intervention can provide in total and subgroups stratified by the individual’s level of cognitive performance (CPS2 = 1–4).

Needed services	Total = 278 N (%)	CPS2 = 1 N (%)	CPS2 = 2 N (%)	CPS2 = 3 N (%)	CPS2 = 4 N (%)	*P*
Knowing what changes to expect and how to adapt	184(66.2)	7(53.8)	38(55.1)	98(71.0)	41(70.7)	0.02*
Proactive planning for future, e.g., financial plan.	167(60.1)	5(38.5)	36(52.2)	85(61.6)	41(70.7)	0.067
Regularly talking to a professional counselor	165(59.4)	5(38.5)	36(52.2)	85(61.6)	39(67.2)	0.085
Accessing information/support for you as a caretaker/caregiver	162(58.3)	6(46.2)	37(53.6)	83(60.1)	36(62.1)	0.511
Other family members or friends or neighbors understand the condition of the individual with cognitive problems and your needs	151(54.3)	5(38.5)	35(50.7)	77(55.8)	34(58.6)	0.103
Telephone/email consultation on request over entire disease course	147(52.9)	4(30.8)	29(42.0)	78(56.5)	36(62.1)	0.03*
Knowing potential support that other family members or friends or neighbors can provide	143(51.4)	3(23.1)	34(49.3)	77(55.8)	29(50.0)	0.076
Knowing how to solve time conflicts for family meetings	141(50.7)	2(15.4)	29(42.0)	79(57.2)	31(53.4)	0.000***
Talking to peer groups	142(51.1)	5(38.5)	28(40.6)	73(52.9)	36(62.1)	0.070
Knowing how to communicate with the individual with cognitive problems about the changes	142(51.1)	3(23.1)	33(47.8)	73(52.9)	33(56.9)	0.03*
Knowing how to cope with changes in relationship	142(51.1)	7(53.8)	26(37.7)	74(53.6)	35(60.3)	0.007**
Other family members or friends or neighbors talk to a professional counselor	133(47.8)	3(23.1)	31(44.9)	68(49.3)	31(53.4)	0.497
Knowing reliable information on the condition of the individual with cognitive problems	131(47.1)	5(38.5)	28(40.6)	65(47.1)	33(56.9)	0.01*
Family meetings to talk about the condition, future plan, etc. of the individual with cognitive problems	131(47.1)	3(23.1)	31(44.9)	73(52.9)	24(41.4)	0.03*

Notes: Ratings of these items were made on a 5-point Likert scale (1 = Not applicable, 2 = Satisfied, 3 = Low need, 4 = Need, 5 = High need). Significance:

***p < .001

**p < .01

*p < .05.

‘Telephone/email consultation on request over entire disease course’, ‘Knowing how to communicate with the individual with cognitive problems about the unusual changes’, and ‘Knowing reliable information on the condition of the individual with cognitive problems’ showed an increase in proportion to the severity of cognitive performance. The most needed service, felt by more than half of all participants across subgroups, namely ‘Knowing what changes to expect and how to adapt (53.8%, 55.1%, 71.0%, 70.7% respectively).

In the intact2 (CPS2 = 1) subgroup, two other frequently needed services were ‘Knowing how to cope with changes in relationship’ (53.8%), and ‘Accessing information/support for you as a caretaker/caregiver’ (46.2%).The least frequently needed services, which also shown a great difference from the borderline1 (CPS2 = 2) to more severe subgroups, were ‘Knowing how to communicate with the individual with cognitive problems about the unusual changes’ (23.1% vs 47.8%), and ‘Knowing how to solve time conflicts for family meetings’ (15.4% vs 42.0%).

In the borderline1 (CPS2 = 2) subgroup, three other frequently needed services were ‘Accessing information/support for you as a caretaker/caregiver’ (53.6%), ‘Proactive planning for future, e.g., financial plan’ and ‘Regularly talking to a professional counselor’ (both were 52.2%). The least needed service was ‘Knowing how to cope with changes in relationship’, which was even lower than the intact2 (CPS2 = 1) subgroup (37.7% vs 53.8%).

In the borderline2 (CPS2 = 3) subgroup, three other frequently needed services were ‘Accessing information/support for you as a caretaker/caregiver’ (60.1%), ‘Regularly talking to a professional counselor’ and ‘Proactive planning for future, e.g., financial plan’ (both were 61.6%). The least frequently needed service was ‘Knowing reliable information on the condition of the individual with cognitive problems’ (47.1%). In addition, the percentages of needed services of ‘Knowing how to solve time conflicts for family meetings’ were higher than the subgroup with moderately impaired1 cognitive performance (CPS2 = 4) (57.2% vs 53.4%).

In the moderately impaired1 (CPS2 = 4) subgroup, two other frequently needed services were ‘Proactive planning for future, e.g., financial plan’ (70.7%), ‘Regularly talking to a professional counselor’ (67.2%). ‘Telephone/email consultation on request over entire disease course’, ‘Accessing information/support for you as a caretaker/caregiver’ and ‘Talking to peer group’ shared the same percentage (62.1%). The least needed service was ‘Family meetings to talk about the condition, future plan, etc. of the individual with cognitive problems’, which was even lower than borderline2 (CPS2 = 3) subgroup (41.4% vs 52.9%).

Regarding the qualitative data analysis, a total of 97 different statements were written in the open field of the survey. Six statements addressed the survey (e.g., a question about whether the disease runs in the family should be added). Twenty statements dealt with caregiving experiences or the care recipients’ condition (e.g., ‘my father’s situation was getting worse’, ‘I was worried’). Eleven statements explained their relationship to the person who had cognitive problems/dementia (e.g., ‘My grandmother has dementia’, ‘I cared for my ex-partner’). Six statements mentioned that the intervention would be more helpful in their later stage. For example, ‘My mother has Lewi Body dementia in its very early stage. At the moment, we don’t need much help. But as the disease progresses, I would make use of possible additional support–such as the intervention described in this survey.’ Four statements mentioned that the disease ran in his family (e.g., ‘Both father and brother have dementia’). One statement mentioned the stigma against talking about this: ‘There is an awareness that something is wrong, but it cannot be talked or helped because of the taboo.’ The remaining 49 statements of the total 97 statements were summarized into three main themes.

The first theme concerned negative attitudes and disappointment towards medical or home care services, such as hospitals, medical team, doctors/neurologists, home care and day care. Some examples are:

‘It would help a lot if the patient and caregiver were taken seriously or treated less rudely by doctors/neurologists, especially at the beginning of the treatment process.’

‘I was very disappointed that there was no psychosocial support for us when the medical team made the diagnosis of onset dementia. I am very curious about your research and the intervention under development.’

The second theme was about the need of informal caregivers to have timely guidance, training and support. Respondents reported the need for a coach or a person to guide them through the process. Some examples are:

‘There is not much information or support for caregivers who have a relative or friend or neighbor with onset dementia. There should be! These caregivers also need care.’

‘I have the thought that there should really be a coach who guides me through this. Unfortunately, there is no such thing here.’

‘Guidance is a necessity, especially for informal caregivers who work and not entitled to leave the systems. We don’t have time to figure the things out and don’t know where we can get timely help.’

The third theme was about some useful ways to help informal caregivers, such as family groups, information meetings, and as the reference person for dementia, which can be helpful. Some examples are:

‘I have already attended several meetings about dementia in the past when my dad was demented. Now my uncle also has dementia. Thanks to the information evenings, I have a lot of knowledge and I am well aware of the course of the disease.’

‘I participated in the family group and can contact the dementia reference person in the rest home, which is very important to me. My children are also very involved and supportive.’

## Discussion

A feasibility study is necessary in order to avoid waste of resources for intervention adaptation and evaluation, and to learn from potential users whether an intervention’s content can be acceptable and helpful for the target population. This is the first study to explore the feasibility of a Flemish adaptation of the NYUCI around the recruitment and intervention content for informal caregivers of people with early cognitive decline. Furthermore, differences in acceptability and potential helpfulness of the intervention content were also examined across subgroups stratified by the level of early cognitive decline using the interRAI Cognitive Performance Scale (CPS2).

About 49.7% of the total respondents reported that they probably or certainly would participate in the PROACTIVE study. Specifically, about 44% of the informal caregivers of people with early cognitive decline reported that they probably or certainly would participate in the study. This suggests that we could recruit enough target participants for the next stage of study which is the intervention testing and evaluation, since the focus of PROACTIVE will be on informal caregivers of people with early cognitive decline. Considering the representativeness of experimental sample and recruitment bias, we should pay more attention to the identified associated factors, including cognition, co-habitation, education level, and employment status during the recruitment process. For example, a more proactive technique may be conducted to recruit lower education caregiver group to participate in the intervention compared to the university or higher education caregiver group. The associations with poorer cognition [38] and living with the person with cognitive problems were consistent with previous studies [39]. Additionally, misconceptions about early cognitive decline or early onset dementia should also considered as a possible factor according to our results. About 44.1% of our whole sample agreed with the statement that unusual changes of their relatives or friends or neighbors, such as memory problems, are a normal part of ageing. Aligned with this finding, a recent study showed that more people with mild cognitive impairment (MCI) were reported in community surveys than those who sought help from healthcare providers [40]. Other studies also reported that the failure to acknowledge unusual changes as disease was a barrier to the acceptance process [24] and to seeking information and support [25].

Regarding the acceptability of the PROACTIVE intervention content (i.e., 14 types of services), our study suggests that all these services, could meet the needs of almost half of the total respondents who cared for a person with early cognitive decline (CPS2 = 1–4). Our qualitative data analysis results also supported the respondents’ need for guidance, training and information meetings, and perceived helpfulness of participating in a family group meeting. Previous studies also suggest that many caregivers do not take steps to seek help, often because of tensions in the family [41]. They reported the need of interpersonal support, such as help to make other family members understand what the issues of the disease are [42]. The most acceptable and common service across subgroups stratified by the level of early cognitive decline (CPS2 = 1,2,3, and 4) was ‘Knowing what changes to expect and how to adapt’. This is in line with two studies that underlined that an early-stage intervention should aim at the facilitation of the adaptation process and future care [43, 44]. Through comparisons among the four subgroups, we found that CPS2 = 3–4 subgroups had a higher acceptance of the intervention services compared to the CPS2 = 1–2 subgroups. Almost all services were acceptable by over half of respondents from the CPS2 = 3–4 subgroups, but only two services and five services were acceptable by over half of respondents from the CPS2 = 1 and CPS2 = 2 subgroups respectively.

Regarding the potential helpfulness of the entire intervention and intervention core components, only the ‘peer-support group participation’ component was perceived helpful by less half of the respondents who cared for a person with early cognitive decline (CPS2 = 1–4). A previous study found that stigma resulted in caregivers’ refusal to seek support from families or friends [24, 25, 45]. Informal caregivers might want to talk to peer groups, but they may be afraid of the consequences due to stigma, especially in the very early stage of cognitive decline (CPS2 = 1–2). Interestingly, we found that there was a difference between the proportion of CPS2 = 1–4 group who needed to talk to a peer group (51.1%) and who thought peer-group participation would be helpful (47.8%). The reason for this difference can be further studied in future. Through comparisons among the four subgroups, we found that the intervention was perceived helpful by almost three quarters of the respondents from the CPS2 = 3–4 subgroups, compared to 38.5% from the CPS2 = 1 and 58% from the CPS2 = 2 subgroups. The three core intervention components followed the same pattern. This is also consistent with the higher acceptability of the intervention content in CPS2 = 3–4 subgroups. It means that the CPS2 = 3–4 subgroups would be more likely to accept the intervention and thought they could benefit more, and this information should be considered in the further intervention adaptation process. Considering cost-effectiveness issues, a more needed service package might be designed and provided to the CPS2 = 1–2 subgroups based on the results of acceptability of the intervention services.

### Strengths and limitations

The initial version of the PROACTIVE intervention was developed based on an internationally evidence-based intervention (i.e., NYUCI), which has been put into practice in some states of the US and was adapted in many countries. Our study showed that informal caregivers in Flanders had similar needs to those in the original intervention studies and that the intervention could be of value in Flanders, considering the context of the care sectors in the Flemish region. A second strength of the study is the use of the interRAI CPS2, which is sensitive to assessing early cognitive decline. Based on the results of the subgroup analysis by CPS2 = 1–4, we can identify the subgroups who would most accept the intervention content and benefit more to achieve a greater cost-effectiveness. Moreover, we can further the intervention adaptation process based on our study results through stakeholder meetings etc. in the following phases.

The main limitation of the study is that we used a convenience sampling method to recruit participants. The respondents of the sample were not selected at random from the population. In addition, people answering the survey might be the ones most in need of an intervention or most willing to participate. Although we checked the representativeness according to age, gender and other characteristics, this may not be a representative sample for the whole population of informal caregivers in Flanders. Considering the limitation of the sample representativeness, we should be cautious when generalizing to the population in Flanders, Belgium. Another limitation is that we could not make an in-depth qualitative analysis because there was only one open question included in our survey. Given the difference between the few respondents in CPS2 = 1 and other subgroups, we also should be cautious about the related data analysis results.

## Conclusion

This study provides the first evidence of the feasibility, acceptability, and potential helpfulness of a Flemish adaptation of the NYUCI in the context of informal caregivers of older people with early cognitive decline. The recruitment of enough target participants for a subsequent RCT evaluation study is feasible. Identified associated factors should be considered during the recruitment process. Almost all services from the intervention could satisfy the needs of over half of the target caregivers. The PROACTIVE intervention and all core components except ‘peer-support group participation’ were perceived as potentially helpful by most target caregivers. We also found that the CPS2 = 3–4 subgroups showed greater acceptability of the intervention compared to the CPS2 = 1–2 subgroups. It might be more cost-effective to target the CPS2 = 3–4 subgroups for the intervention and further research is needed. Results from this study will inform the further intervention adaption process and evaluation design in the following phases based on the MRC framework.
